# Supplementary material for: MetaRibo-Seq measures translation in microbiomes
Source: Nat Commun. 2020 Jun 29;11:3268. doi: 10.1038/s41467-020-17081-z (PMC7324362; doi:10.1038/s41467-020-17081-z)
Supplement: Supplementary file 10 — Supplementary Data 7 [file 41467_2020_17081_MOESM10_ESM.zip › File2/Confidence_High_Taxonomy/429277_out.krona.html]

Javascript must be enabled to view this page.

members
magnitude
magnitudeUnassigned
count
unassigned
taxon
rank

429277\_out

3

2
superkingdom
3

2
phylum
976

2
class
200643

171549
order
2

family
171552
2

2
838
genus

2

SRS143214\_contig\_number\_2490SRS143509\_contig\_number\_contig-100\_395.34983
652716
species

phylum
1239
1

1
186801
class

186802
order
1

family
541000
1

genus
1263
1


SRS144183\_contig\_number\_32882
1
2293208
species
